# Simultaneous Three-Degrees-of-Freedom Prosthetic Control Based on Linear Regression and Closed-Loop Training Protocol

**DOI:** 10.3390/s24103101

**Published:** 2024-05-13

**Authors:** Carles Igual, Jorge Igual

**Affiliations:** Instituto de Telecomunicaciones y Aplicaciones Multimedia (ITEAM), Universitat Politècnica de València, 46022 Valencia, Spain; carigba@alumni.upv.es

**Keywords:** electromyography, adaptive filter, prosthetics, proportional control, task analysis, psychomotor performance, computer-based training, linear regression

## Abstract

Machine learning-based controllers of prostheses using electromyographic signals have become very popular in the last decade. The regression approach allows a simultaneous and proportional control of the intended movement in a more natural way than the classification approach, where the number of movements is discrete by definition. However, it is not common to find regression-based controllers working for more than two degrees of freedom at the same time. In this paper, we present the application of the adaptive linear regressor in a relatively low-dimensional feature space with only eight sensors to the problem of a simultaneous and proportional control of three degrees of freedom (left–right, up–down and open–close hand movements). We show that a key element usually overlooked in the learning process of the regressor is the training paradigm. We propose a closed-loop procedure, where the human learns how to improve the quality of the generated EMG signals, helping also to obtain a better controller. We apply it to 10 healthy and 3 limb-deficient subjects. Results show that the combination of the multidimensional targets and the open-loop training protocol significantly improve the performance, increasing the average completion rate from 53% to 65% for the most complicated case of simultaneously controlling the three degrees of freedom.

## 1. Introduction

Electromyography (EMG) measures muscle response or electrical activity in response to a nerve’s stimulation of the muscle [1]. Based on the location of the sensors, it can be intramuscular or surface electromyography. Since the intramuscular case requires an implantable device with corresponding risks such as potential physical harm, it is more common to use the EMG registered on the surface of the skin. Although it can only capture EMG signals from multiple groups of muscles and is noisier since different artifacts and crosstalk affect the quality of the registered EMG signal [2], it has been shown that the information acquired is enough for the myoelectric control of prosthetic limbs [3]. In the rest of the paper, the term EMG is used to express the surface EMG.

There are different myoelectric control strategies [4]. The most basic one and common in commercial devices is the on/off strategy. It maps the EMG signal to a switch control of the prosthesis. If the amplitude of the EMG signal is greater than a pre-established threshold, the corresponding movement is activated. This is a very simple control strategy that has the limitation of only controlling one degree of freedom (DoF) at a time. Commercial prostheses try to overcome this limitation with sequential cumbersome switching mechanisms. This DoF admits different actions depending on the mechanical output, e.g, grasp an object or rotate the wrist. In addition, the speed of the movement is constant since this strategy only allows an on/off movement. It can be modified to incorporate a proportional control, where the speed or force of the movement is related to the amplitude of the EMG signal.

The next generation of controllers were inspired by machine learning solutions, initially the pattern recognition or classification approach and later the regression solution. Both strategies are based on the assumption that there is a correspondence between the intended action and the EMG signals. The algorithm must learn this relationship during the training session. The classification approach consists of obtaining a feature vector from the EMG recorded signals (feature engineering) and associating it with the intended movement (pattern recognition and fit a classifier) [5]. It implies that the range of effective movements is reduced since the classifier is trained only for a finite set of patterns. The classification-based controllers have been successfully applied, improving the accuracy of motion intent recognition [6]. One advantage with respect to the switch on/off controllers is that they can be trained for more than one DoF. However, they present some limitations inherent to the discrete nature of the classification statement of the problem. To assume that there are only a few set of movements and that completing actions are independent events is far from the human experience for limb action movements; it is really a much more complicated and dynamic process. In order to approximate the controller functionality to the natural and intuitive human experience, the regression approach tries to learn a continuous range of movements, instead of a predefined set of robotic movements.

The regression approach allows a simultaneous and proportional controller, i.e., the estimation of continuous human motor upper limb intention based on EMG signals [7]. The difficulty comes from the fact that it is more complicated to find a mapping for a continuous than for a discrete set of movements. Considering that the EMG signals are non-stationary and can be affected by a large number of exogenous variables such as fatigue, changes in conductivity, and so on, it is not strange that the machine learning solutions have not yet been implemented as the common solution in real-world prostheses. The reliability of the on/off strategy weighs more than the possibility of obtaining a more intuitive and natural controller with more DoFs. Recent academic research on using deep learning solutions can be helpful to overcome some limitations of traditional machine learning solutions since they allow to encode deeper relationships, but they are even further from being intuitive and they can overwhelmingly complicate the model [8]. The lack of explainability also does not help the deep learning strategies.

One advantage of the regression strategy is its ability to mathematically model the natural human experience [9]. For example, we can model a velocity control experience just introducing a recursive term in the estimation of the intended movement. In other words, it is straightforward to model time dependencies that in the traditional supervised classification approach are not considered and in the deep learning paradigm will require even more complicated models, such as recursive neural networks and variants that require a huge amount of training data [10]. It is important to note that in the end, each person using a prosthesis must undergo a learning/practicing process to be familiar with the prosthesis and the controller. It is unrealistic to think that this training process is compatible with today’s deep learning procedures. It will require some kind of transfer learning from a universal user that is not possible due to very different anatomies, limb deficiencies, and many other reasons that make it not possible to find a unique controller solution for every person. Even in that case, the training sessions would be too lengthy and tiring for patients.

From the point of view of the number of degrees of freedom, the classification approach requires that the number of targets to be trained are increased exponentially. It complicates the training sessions and reduces the effectiveness of the classifier for the same amount of data. This is the reason that it is not very common to find papers with more than two DoFs at the same time. Additionally, while a lot of attention is on obtaining a robust, adaptive, and reliable motion estimation using machine and deep learning (see survey [11] for a detailed analysis of recent advances in multimodal sensing fusion, transfer learning and post-processing), little attention is given to the training experience. As mentioned in [12], an ideal prosthesis control should require an intuitive, closed-loop, adaptive, and robust to real-time EMG-based interface. In [13], we present a new training paradigm that uses a closed-loop and intuitive training model in order to obtain a better motion controller in a real-time adaptive training session.

To the best of our best knowledge, no previous work using the basic linear regression adaptive filter approach in a low-dimensional feature space has been published showing results in a virtual task environment for more than two DoFs. In this paper, we will address this issue and also another related one: the training strategy. Since the number of simultaneous DoFs is increased while capturing the same information (the same low-dimension feature vector as in the standard two-DoFs case), it is very important to analyze if some training strategies are better than others during the learning process, so the increase is the complexity of the problem (a new set of coefficients must be estimated for the third DoF) and can be compensated for by a better training procedure.

In the next Section 2, we review some related works; in Section 3, we present the study design, the data collection procedure, the regressor controller, the different training paradigms, and the test experiment and metrics to compare the performance results. In Section 4, we show the results obtained and discuss them.

## 2. Related Works

One of the first works to extract simultaneous and proportional control information for multiple (more than two) DoFs under a pattern recognition strategy was performed by Jiang et al. [14]. It was extended by Muceli and Farina [15] up to four wrist DoFs (wrist flexion–extension, radial–ulnar deviation, forearm pronation–supination, and hand closing) using an artificial neural network. The training protocol included 7 one-DoF movements, including the rest position and 14 two-DoF movements, as a combination of the previous ones. Due to the complexity of the classification model, it required a large number of electrodes (in the range 86–97, depending on the subject) and was tested in six able-bodied subjects.

In recent years, related works following the same classification approach have been published. They differ in the classifier, the dimensions and nature of the feature vector, and the number and kind of tested subjects. Recent advances in problems with a large number of DoFs such as hand movements are based on the deep learning approach, e.g., in [16], Simpetru et al. present an accurate continuous prediction of 14 DoFs of the hand using digital cameras and a high-density EMG from the forearm and wrist muscles. An alternative approach is to use a neural-driven musculoskeletal model to predict multiple DoFs [17], in this case metacarpophalangeal joint flexion–extension and wrist joint flexion–extension. Again, a large number of sensors is required to obtain an informative EMG (four 64-channel electrode grids attached to four forearm muscles), and it was only tested in non-disabled subjects.

In the case of regression-based solutions, Li et al. [18] recently implemented a regression-based EMG control method, which achieved the simultaneous and proportional control of two DoFs in a virtual task. They used the best electrodes from a pool of 16 and tested two different 2-DoFs scenarios: hand open–close and wrist pronation–supination controlled virtual target size and rotation, respectively, and wrist flexion–extension and ulnar–radial deviation controlled virtual target left–right and up–down movement, respectively. They tested in 11 able-bodied and 4 limb-absent participants in virtual target matching and fixed targets. Their results showed the feasibility of 2-DoFs simultaneous proportional myoelectric control as it has been reported in other previous works [19,20,21]. The regression approach can be extended to include other non-EMG signals. In [22], Mao et al. proposed a new control scheme that simultaneously estimates continuous grip force and wrist angles using a combination of EMG and acceleration signals. They trained and compared several non-linear regressors based on the SVM classifier using four different EMG features sets in combination with accelerated signals in nine intact subjects.

## 3. Materials and Methods

A human-prosthesis collaboration system devoted to predicting a continuous prosthetic movement based on EMG signals consists of a human, EMG acquisition, preprocessing and feature extraction, an intention prediction algorithm, a control system, and tasks to be executed (application). In the next subsections, we describe the different components of the system with special emphasis on the different training paradigms used to learn the corresponding model.

### 3.1. Subjects

Ten able-bodied participants (4 females and 6 males) and three amputees (1 female and 2 males) were involved in this experiment. Participants were in between 24 and 40 years old. All individuals provided written informed consent before the experiment. The experiments were performed in accordance with the declaration of Helsinki and were approved by the UPV ethics committee (approval number P11-23-03-18).

### 3.2. Data Acquisition and Feature Extraction

The EMG data were recorded with a Myo Armband (Thalmic Labs, Waterloo, ON, Canada). It is a popular device used in similar research works [23]. The armband includes eight bipolar EMG electrodes sampling at 200 Hz wearable by participants with a 19 to 33 cm forearm circumference. The sensor was connected via Bluetooth to a 2.6 GHz personal laptop with 8 GB RAM for data acquisition and processing using a customized framework in MATLAB version: 9.13 (R2022a) (The MathWorks Inc., Natick, MA, USA).

The EMG data were sampled at $f_{s}=200$ Hz, and for the feature vector x(t), the root mean square (RMS) was extracted for each channel from sliding windows of T=200 ms with an increment of 40 ms. For able-bodied participants, the armband was always placed on the right forearm (all of them were right handed), while for participants with limb deficiencies, it was placed on the affected side. The location and orientation of the sensor were consistent for all participants, targeting the flexor carpi radialis and ulnaris muscles, extensor carpi radialis longus and the brachioradialis muscle, among other residual EMG measurements.

### 3.3. Control Model

A linear regression model was used to control three independent degrees of freedom (DoFs) in the position control mode. Each DoF was controlled by an independent model. The three DoF tested motions were wrist flexion–extension, ulnar and radial deviation, and hand open–close. With the regression-based model, it was possible to perform all movements simultaneously and independently in the time and activation ratio.

The regression model maps the extracted features x(t) into a 3D position vector y(t); mathematically, it reads

(1) $$y_{i}\left(t\right)=\sum_{j=1}^{M}b_{i,j}\left(t\right)x_{j}\left(t\right),\;i=1,2,3$$

where $y_{i}\left(t\right),i=1,2,3$ is the current position in each DoF, M=8 is the number of myoelectric channels (electrodes), and $b_{i,k}\left(t\right)$ are the coefficients to be estimated. This model is based in our previous work [20] with q=p=0, where *q* are the tabs of a moving-average (MA) filter related to x(t), and *p* are the filter tabs related to y(t) (see [20] for further details). The goal is to find out the coefficients $b_{i}\left(t\right)$ that minimize the mean squared error.

### 3.4. Study Design

The purpose of the experiment was to test the effects of closed-loop co-adaptive training with real-time feedback to the user against a classical blind open-loop training protocol. In an open-loop training, the goal is to find the coefficients $b_{i,j}\left(t\right)$ that minimize the mean squared error between the real target position $d_{i}\left(t\right)$ and the estimated position $y_{i}\left(t\right)$ with no visual feedback for the participant; they simply know that when performing a given EMG pattern, the algorithm will be able to translate it to the intended movement, e.g., upper right and closing.

On the contrary, the closed-loop co-adaptive learning strategy used in this study allows to take into account the dynamics of the process generating an interaction between the user and the machine during the training phase. While training, the human and the machine have real-time feedback from one another in both directions, not only the algorithm in the controller. Therefore, both agents are able to interact and respond dynamically to the learning of the other, adapting faster and allowing the system to avoid local minimum solutions. This interactive training procedure makes each training session personal and non-transferable, so the controller can only be trained online at the same time that the participant is running the training experiment (in the open-loop strategy, the controller can be retrained later offline using the set of recorded EMG target pairs). For more details about the importance of the closed-loop training procedure, see [13]. In the next subsection, we provide the details about the different computer-based training paradigms following the typical visual cue approach [24].

### 3.5. Training Paradigms

The computer user interface represented a three-dimensional environment. The three controllable DoFs were the 2D position of an moving object and its size. The vertical and horizontal axes were related to the flexion–extension and radial–ulnar deviation of the wrist, respectively, while the opening and closing of the hand controlled the size of the object.

**Figure 1:**
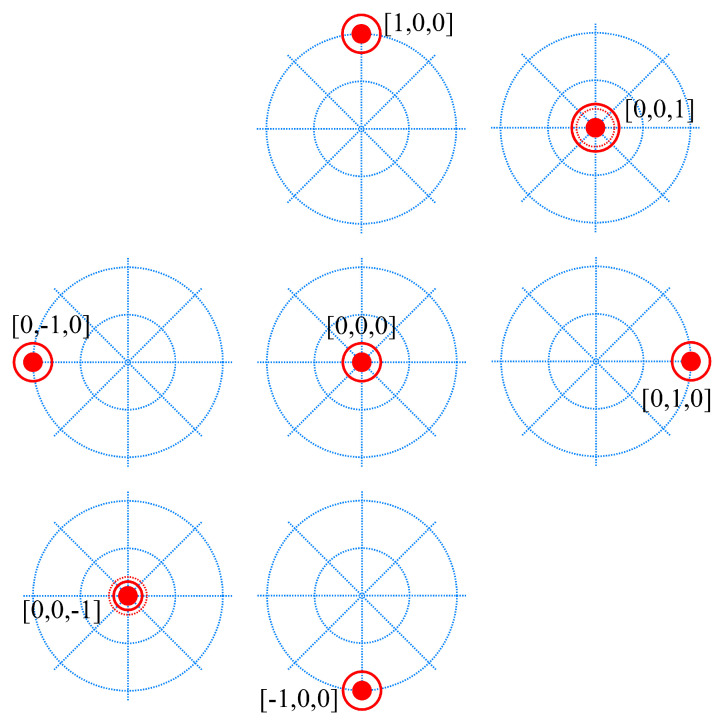
Blind training 1 active DoF targets. The controllable dimensions are the x and y axes and the size of the target. The first two dimensions are represented by a solid circle located at the exact position. The third dimension is represented by a solid ring that increases or decreases its size. To give feedback of the neutral position in the third dimension, the target element has a dashed ring in the 0 value of the third dimension.

During the training, the targets were labeled depending on their number of active DoFs. The target values on each DoF were set to −1, 0 or 1 (−1 and 1 mean that the corresponding DoF is active, while 0 sets the DoF to an inactive condition). This results in 27 possible targets, combining the three possible values at each DoF: rest position [0, 0, 0], 1 active DoF (6 possible targets), 2 active DoFs (12 possible targets), and all 3 DoFs active (8 possible targets). Representing the 3-DoFs combinations in vector notation [flexion–extension, radial–ulnar, opening–closing], Figure 1 shows the six different cases when only one DoF is active [1, 0, 0], [−1, 0, 0], [0, 1, 0], [0, −1, 0], [0, 0, 1] and [0, 0, −1], in addition to the rest position [0, 0, 0] case. Note how the location of the target is moved from the center when the horizontal or vertical movements are active (only one at the same time). In these cases, the size of the target is the same at all locations since the opening/closing DoF is not active. However, when the opening/closing D0F is active, the target stays in the center since the other DoFs are inactive (remember that the figure represents only 1-DoF active situations) and is represented by changing the size of the target.

**Figure 2:**
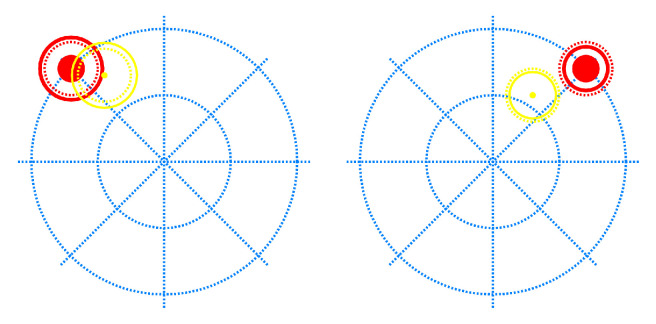
Feedback training at 3-DoFs experiment. The targets follow the same representation as in Figure 1. The user estimate is represented by a yellow object that follows the same principles. In this case, there is a pointer that marks the location of the two first dimensions and should be fit into the solid area of the target. The controllable object also has a ring whose size should match that of the target.

Following the strategy explained in the previous subsection, we designed three different training protocols and compared the real-time control performance achieved with each of them:

- Protocol I (base training): blind training with no user feedback. A lap is defined as going once through all the possible 1 active DoF targets with a rest target after each of them (Figure 1). One training session with this protocol consists of five laps. Only 1-DoF targets are trained at the same time.
- Protocol II (open-loop training): blind training with no user feedback. It starts with one lap as defined in Protocol I. Afterwards, all possible combined targets (2 and 3 active DoFs) are included once, with a rest target after each of them. The difference with respect to Protocol I is that here, the training goes through combined DoFs targets to reduce the non-trained regions. No feedback is given to the user during the training session. They must focus on being consistent, providing the corresponding EMG signal while the trained target is the same.
- Protocol III (closed-loop training): it builds up one blind lap as defined in Protocol I. Afterwards, we activate the user feedback showing the controller output with another mobile element (Figure 2). The training continues, including all the possible combined targets (2 and 3 active DoFs) once, with a rest target after each of them. The difference with respect to Protocol II is that the subject has feedback during the combined DoFs targets.

The training targets were shown in the same pseudo-random order for all participants at each protocol.

### 3.6. Test Paradigm

After each training, the participants performed a test where they had to fit the cursor into a positional target. By controlling the 3D cursor, they had 20 s to reach each target’s 3D position with less than 10% error measuring the Euclidean distance in the 3D space. If they were capable of reaching and holding this position for 1 s within the 20 s, the target was considered a hit. If this requirement was not fulfilled, the next target was prompted. The test was composed of 27 targets, the 26 possible targets combination of the three DoFs plus the rest position, covering the complete output space. As in the training phase, the test targets were pseudo-randomized and shown in the same order for all participants.

### 3.7. Metrics

Classification accuracy is the main metric used to analyze the general performance of a classification-based controller [25]. In our case, with the regression problem, since the predicted and target values are continuous variables, it is common to use the mean square error, i.e., the average difference between the squared of the predicted value by the controller and true position at some time or related metrics such as the root mean square error, correlation coefficient, or $R^{2}$ values [26]. However, these metrics are not so useful in our case since they do not provide a description of the performance of the complete system in terms of real-time task accuracy and efficiency. Different from these typical error-based metrics, performance metrics measure the accuracy of the complete system in real time in different ways [27,28]. In spite of recent efforts [29] to value the offline evaluation, to measure the real-time performance is much more relevant [30]. We will calculate the following ones:

- Completion rate (CR): number of hits (targets held for 1 s within 20 s) over the total amount of test targets (27). A large value means that the controller is able to hit most targets.
- Path efficiency (PE): the shortest distance between targets divided by the user traveled distance. A small value means that the subject is able to reach the target in a fast way, close to the human experience.
- Attempt ratio (AR): the ratio between the number of target entrances during the test and the number of hit targets (this measures the average entrances needed to hit a target). A small value means that the target was hit more easily.

## 4. Results

**Figure 3:**
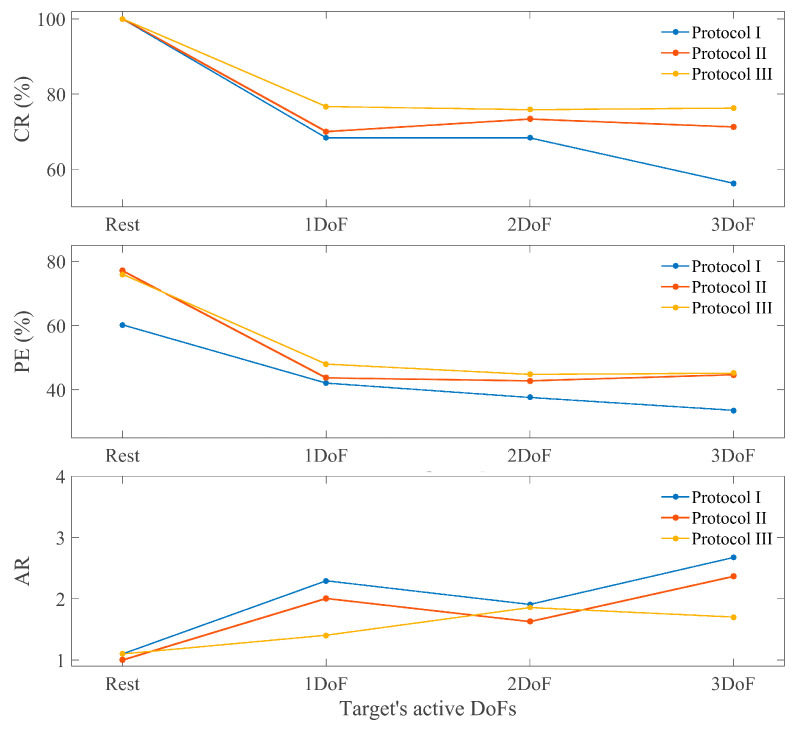
Able-bodied metrics. Each panel shows the results for each metric depending on the type of target and the training protocol used. Protocol I: blind training with 1 active DOF targets, Protocol II: blind training with 3 active DoF targets, Protocol III: co-adaptive training with 3 active DoF targets.

**Figure 4:**
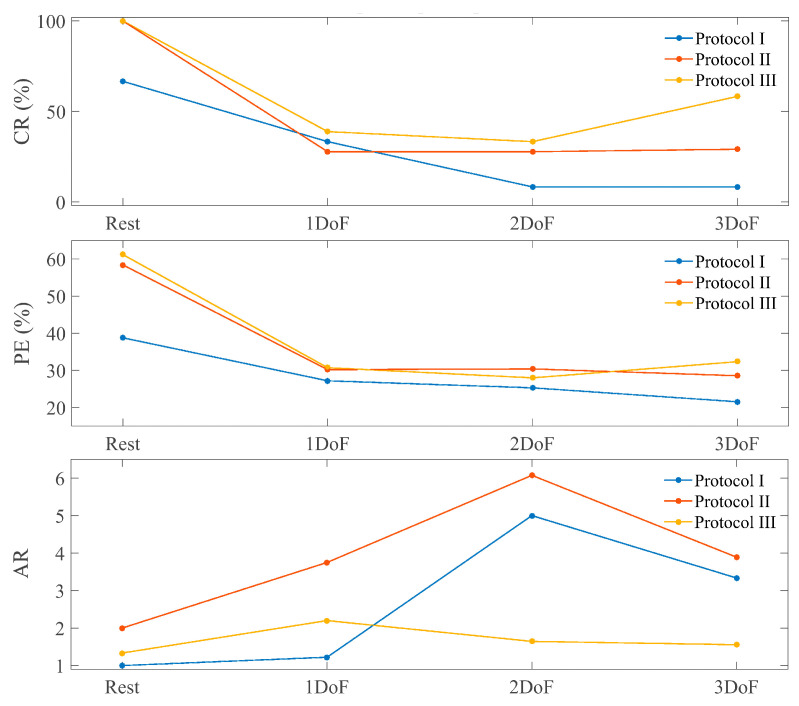
Metrics of participants with limb deficiencies. Each panel shows the results for each metric depending on the type of target and the training protocol used. Protocol I: blind training with 1 active DOF targets, Protocol II: blind training with 3 active DoF targets, Protocol III: co-adaptive training with 3 active DoF targets.

Figure 3 shows the CR, PR (in percentage), and the AR for able-bodied participants for the different training protocols and test targets. Figure 4 shows the same metrics for the group of subjects with limb deficiencies.

The results show an improvement in all metrics from Protocol I to Protocol II (except at the AR for amputees) as a consequence of introducing the combined targets in the blind training. Protocol III achieves the best results globally for each type of target and subject and on average (Figure 3 and Figure 4), proving the utility of the co-adaptive approach.

**Figure 5:**
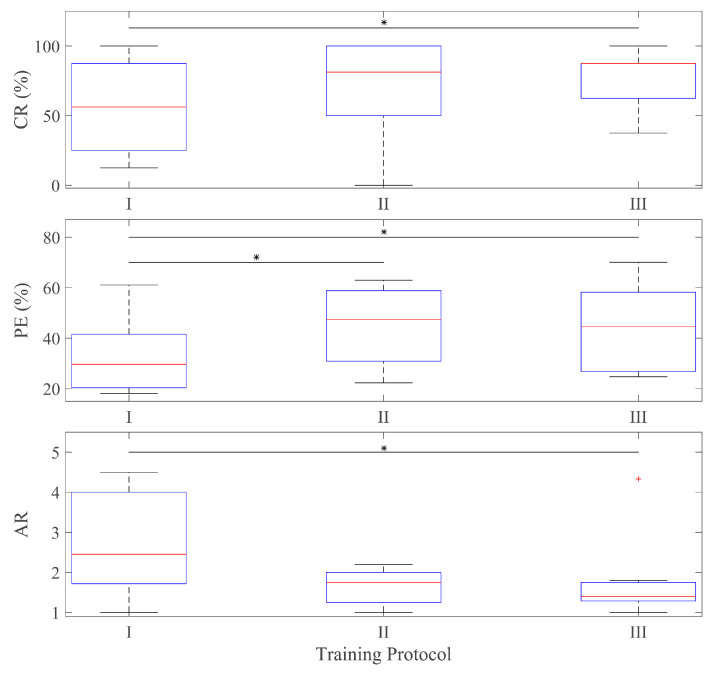
Statistical test. Each panel shows the boxplots (the bottom and top edges of the box indicate the 25th and 75th percentiles, respectively; the red line indicates the median and outliers are plotted individually using the red + symbol.) for each metric in the case of 3 active DoFs targets (only with able-bodied participants, as the number of participants with limb deficiency is not large enough for a statistical test). The cases with significant difference are indicated with an asterisk *. Protocol I: blind training with 1 active DOF targets, Protocol II: blind training with 3 active DoF targets, Protocol III: co-adaptive training with 3 active DoF targets.

A non-parametric Friedman test revealed that the improvement from training with 3-DoF targets vs. 1-DoF targets (Protocol I vs. II) was statistically significant only for path efficiency (Figure 5). However, the co-adaptive training (Protocol III) was statistical significant better than Protocol I for all three metrics. In the case of PE, the significance was extended to 2 active DoFs targets. This proves how Protocol III is significantly better than Protocol I for the goal of controlling simultaneously the 3 DoFs while none of them remains inactive.

**Figure 6:**
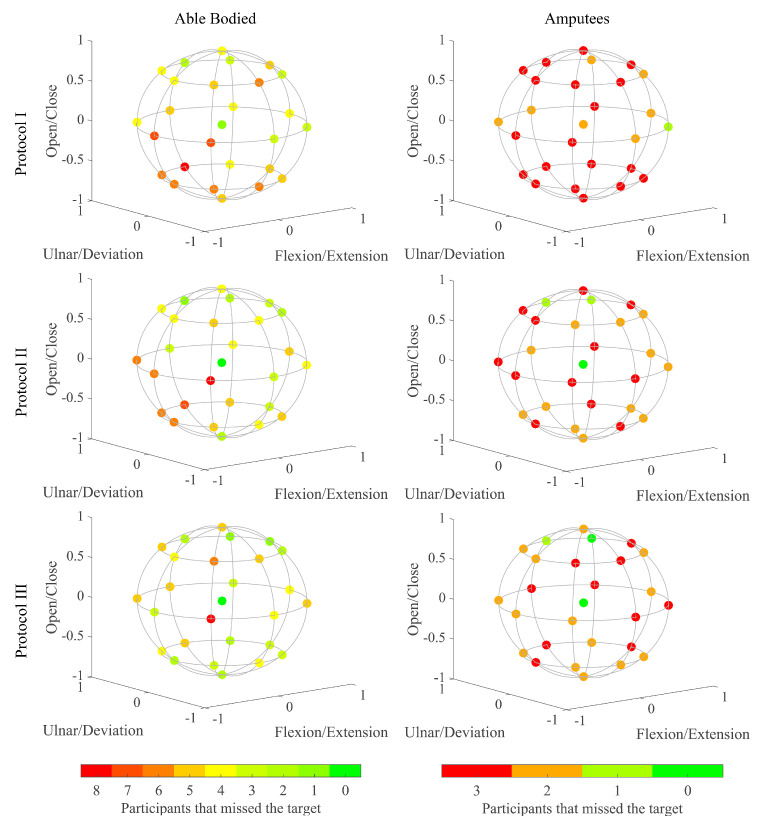
Completed targets. The plot is divided depending on the participant group and the training protocol used. Each target is colored depending on the number of participants that missed it. Protocol I: blind training with 1 active DOF targets, Protocol II: blind training with 3 active DoF targets, Protocol III: co-adaptive training with 3 active DoF targets.

In Figure 6, we visualize the performance for each of the 1-, 2- and 3-DoF targets individually, focusing on the Completion Rate metric. For able-bodied participants, there is a positive trend as we progress with the training protocols. We can see how the most problematic region is the flexion combined with ulnar deviation and closed hand (predominated by orange targets). We see how Protocol III improves this region, especially for able-bodied participants, with the appearance of more green targets, reaching 70% success among participants. We see again a similar behavior for amputees; however, in this case it is extended to the entire flexion region. The region is predominated by red targets turning into orange at Protocol III, also showing some green targets in the open hand, radial deviation and extension region.

All these results prove how the introduction of multiple active DoFs targets combined with co-adaptive learning allow the model to improve the learning of a fully simultaneous and proportional 3D control for able-bodied participants. Adding only one of the two attributes to the training did not generate a significant improvement, while the combination of both proved to be the key to a useful control. So far, 3D control has been solved by controlling the 3 DoFs in pairs, always leaving one inactive and switching the combined pair. The novelty here relies on the capability to fully control the 3 DoFs at the same time, achieving a consistent performance.

## 5. Discussion and Conclusions

In the works where more than two DoFs are considered, it is common to switch between pairs of DoFs and have an external model to activate each pair. In this way, the training of the controller can be simplified since only a two-DoFs prediction has to be made at any time. Here, we have presented for the first time an experiment for a fully simultaneous and proportional EMG control with three DoFs, including results for three real patients, not only healthy subjects. The most important result is that adding combined targets or co-adaptation does not generate significant results but both together do. However, a simultaneous and proportional 3-DoFs task is still a hard one, especially for the limb-deficient group. Since it is so difficult to recruit this kind of patient in real studies, we cannot run a deep statistical analysis nor study how the origin of the limb deficiency can affect the results, e.g., for amputees, whether the subject has to learn from zero or they are still familiar with the muscle movements before their amputation. Nevertheless, the improvement in the results for this group when combining the closed-loop training with multidimensional targets shows a promising future for this idea of including some feedback during the training so that not only the machine is able to learn but the user also learns how to generate a better EMG signal. As far as we know, these are the best results obtained for this group of people so far using a low-dimensional feature space (only eight sensors) linear regressor. In future work, we will further analyze the influence of physiological measures such as arm length and explore new user interfaces based on augmented reality by trying to visually simulate a prosthetic hand. This can be very helpful, especially for the case of limb-deficient people since they have no visual intuitive feedback during the experiment but the visual cue. The visualization of an animated hand in a realistic three-dimensional space is expected to improve the performance of this group of people.

## Data Availability

Data are contained within the article.
